# Cancer Cell Metabolism Bolsters Immunotherapy Resistance by Promoting an Immunosuppressive Tumor Microenvironment

**DOI:** 10.3389/fonc.2020.01197

**Published:** 2020-07-22

**Authors:** Zhou Jiang, Jennifer L. Hsu, Yintao Li, Gabriel N. Hortobagyi, Mien-Chie Hung

**Affiliations:** ^1^Department of Molecular and Cellular Oncology, The University of Texas MD Anderson Cancer Center, Houston, TX, United States; ^2^Department of Breast Medical Oncology, The University of Texas MD Anderson Cancer Center, Houston, TX, United States; ^3^Center for Molecular Medicine and Research Center for Cancer Biology, Graduate Institute of Biomedical Sciences, China Medical University, Taichung, Taiwan; ^4^Department of Biotechnology, Asia University, Taichung, Taiwan

**Keywords:** immune checkpoint inhibitors, cancer metabolism, tumor microenvironment, immune evasion, cancer cell metabolite

## Abstract

Immune checkpoint inhibitors (ICIs) targeting immune checkpoint proteins, such as CTLA-4 and PD-1/PD-L1, have demonstrated remarkable and durable clinical responses in various cancer types. However, a considerable number of patients receiving ICIs eventually experience a relapse due to diverse resistance mechanisms. As a result, there have been increasing research efforts to elucidate the molecular mechanisms behind resistance to ICIs and improve patient outcomes. There is growing evidence that the dysregulated metabolic activity of tumor cells generates an immunosuppressive tumor microenvironment (TME) that orchestrates an impaired anti-tumor immune response. Notably, the immunosuppressive TME is characterized by nutrient shortage, hypoxia, an acidic extracellular milieu, and abundant immunosuppressive molecules. A detailed understanding of the TME remains a major challenge in mounting a more effective anti-tumor immune response. Herein, we discuss how tumor cells reprogram metabolism to modulate a pro-tumor TME, driving disease progression and immune evasion; in particular, we highlight potential approaches to target metabolic vulnerabilities in the context of anti-tumor immunotherapy.

## Introduction

Immune checkpoint proteins are paired regulators of immune response. These molecules are crucial for self-tolerance, which blocks immune cells from attacking autologous cells aimlessly. However, by utilizing immune checkpoint pathways, cancer cells can send an “off” signal to anti-tumor immune cells and escape immune surveillance. Immunotherapy drugs called immune checkpoint inhibitors (ICIs) work by blocking checkpoint proteins from binding with their partner proteins, and this in turn decreases the “off” signal and enhances anti-tumor immunity. Immunotherapy promises to be more significant than any other form of treatment, especially for patients whose tumors have already metastasized. Two pathways that control immune inhibitory signals have been successfully targeted in clinical applications: (1) cytotoxic T-lymphocyte-associated protein 4 (CTLA-4) and its ligand B7 molecules and (2) programmed cell death 1 (PD-1) and programmed cell death ligand 1 (PD-L1) (1, 2). Antibodies against CTLA-4, PD-1, and PD-L1 have demonstrated durable clinical responses and have been approved by the U.S. Food and Drug Administration for various cancers (Table 1).

**Table 1:** U.S. Food and Drug Administration–approved immune checkpoint inhibitors for cancer treatment.

**Name**	**Target**	**Indications**	**Approval date**
Ipilimumab	CTLA-4	Melanoma	Mar 2011
Pembrolizumab	PD-1	Melanoma	Sep 2014
		NSCLC	Oct 2015
		Head and neck cancer	Aug 2016
		Hodgkin lymphoma	Mar 2017
		MSI-H/dMMR	May 2017
		Bladder cancer	May 2017
		Gastric cancer	Sep 2017
		Cervical cancer	Jun 2018
		PMBCL	Jun 2018
		Hepatocellular carcinoma	Nov 2018
		Merkel cell carcinoma	Dec 2018
		Renal cell carcinoma	Apr 2019
		SCLC	Jun 2019
		Esophagus cancer	Jul 2019
		Endometrial carcinoma	Sep 2019
Nivolumab	PD-1	Melanoma	Dec 2014
		NSCLC	Mar 2015
		Renal cell carcinoma	Nov 2015
		Hodgkin lymphoma	May 2016
		Head and neck cancer	Nov 2016
		Bladder cancer	Feb 2017
		Colorectal cancer	Aug 2017
		Hepatocellular carcinoma	Sep 2017
		SCLC	Aug 2018
Cemiplimab	PD-1	SCC	Sep 2018
Atezolizumab	PD-L1	NSCLC	Oct 2016
		Bladder cancer	May 2016
		SCLC	Mar 2019
		Breast cancer	Mar 2019
Durvalumab	PD-L1	Bladder cancer	Feb 2016
		NSCLC	Feb 2018
		SCLC	Mar 2020
Avelumab	PD-L1	Merkel cell carcinoma	Mar 2017
		Renal cell carcinoma	May 2019
Ipilimumab+nivolumab	CTLA-4+PD-1	Melanoma	Oct 2015
		Renal cell carcinoma	Apr 2018
		Colorectal cancer	Jul 2018
		Hepatocellular carcinoma	Mar 2020

*Data from U.S. Food and Drug Administration website (before March 2020). MSI-H, high levels of microsatellite; dMMR, deficient mismatch repair; PMBCL, primary mediastinal B-cell lymphoma; SCLC, small cell lung cancer; SCC, squamous cell carcinoma*.

Despite the promising clinical success of ICIs, ~60 to 70% of patients do not respond to immunotherapy as a single agent; in contrast, those who demonstrate initial response eventually develop resistance (3). A number of mechanisms of resistance to ICIs, including interferon (IFN)-γ signaling pathway mutations, JAK1/JAK2-inactivating mutations, the absence of antigen presentation, and the upregulation of IFN pathway-driven inhibitory immune checkpoints, have been well-investigated (4, 5). In addition, in the tumor microenvironment (TME), mounting evidence indicates that abnormal metabolic activities of cancer cells play an essential role in the suppression of the anti-tumor immune response and lead to tumor immune evasion and metastasis (6).

Therefore, a deep understanding of the metabolic differences between tumor and normal tissue and their impact on anti-tumor immune response will not only help expand therapeutic options, but also help overcome the issue of resistance over time. The impact of metabolism on the tumor cell itself, including the generation of redox equivalents, energy, and macromolecules (proteins, lipids, DNA, and RNA) has been well-addressed (7). In this review, we focus on how the abnormal metabolism of tumor cells modulates the TME profile and leads to tumor progression and immune evasion. Furthermore, on the basis of current knowledge of cancer cell metabolism, we highlight potential therapeutic strategies that could restrict tumor progression and enhance the anti-tumor effect of ICIs.

## Tumor Metabolic Stress Shapes an Immunosuppressive TME

### Nutrient Competition

**Figure 1:**
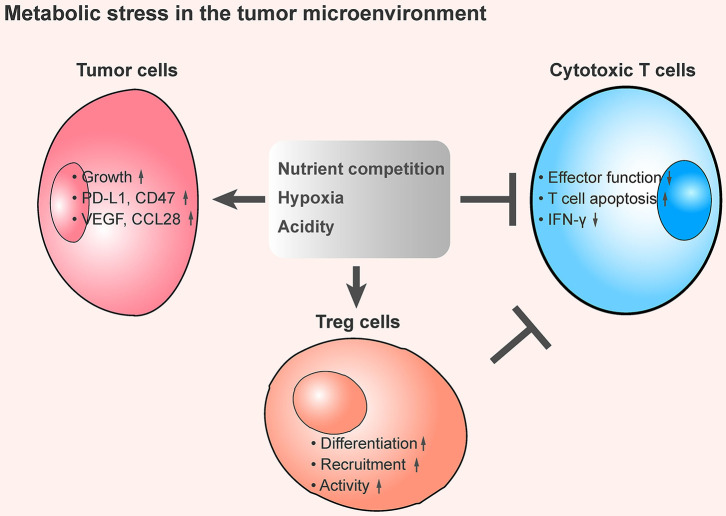
The tumor metabolic stress shapes an immunosuppressive the tumor microenvironment. An overview of metabolic stress in the TME that mediates immune suppression. In the TME, cancer cells exhibit a substantial demand of nutrients, including glucose, amino acids, and fatty acids, and this contributes to the lack of dioxygen and maintains high production of H^+^. These metabolic stresses promote tumor cell growth, increase the expression of immune checkpoint proteins and immunosuppressive cytokines secretion, enhance the inhibitory function of regulatory T cells, and inhibit the anti-tumor effect of tumor-infiltrating cytotoxic T cells, thereby leading to an immunosuppressive TME.

Nutrient competition between different cells can influence cell survival, growth, and function. The demand for nutrients is especially high in the TME, and the competition between tumor cells and immune cells can dampen the anti-tumor response (Figure 1).

Anti-tumor immunity is impaired by the competition for carbohydrates. Tumors exhibit high rates of glycolysis; thus, glucose is in high demand in tumor cells. One study showed that upregulated PD-L1 on tumor cells promotes mammalian target of rapamycin (mTOR) activity and glycolytic metabolism, which leads to tumor-mediated glucose restriction, alters CD8^+^ T-cell metabolism, and dampens the ability of T cells to produce IFN-γ. Even when tumors are highly antigenic, the competition for glucose still inhibits T-cell activity (8). Glucose deprivation also suppresses anti-tumor effector functions of intratumoral Th1 CD4^+^ T cells by limiting the Ca^2+^-NF-AT1 signaling pathway in CD4^+^ T cells (9).

Also damaging to anti-tumor response is the competition for amino acids. The enzyme indoleamine 2,3-dioxygenase (IDO), which catalyzes the essential amino acid tryptophan along the kynurenine pathway, is widely expressed in human cancers, and higher IDO expression is correlated with poorer prognosis in a variety of cancer types (10). Expression of IDO by tumor cells, dendritic cells, and macrophages leads to immune suppression within the TME (11–14). IDO contributes to immune regulation by producing kynurenine, a ligand for the aryl hydrocarbon receptor, and by consuming tryptophan to trigger amino acid-sensing signal transduction pathways. The upregulated kynurenine activates the aryl hydrocarbon receptor, which is a ligand-activated transcription factor (15), and increases immunosuppression by promoting differentiation of T-regulatory cells (Tregs) (16), and this in turn suppresses anti-tumor immune response (17) and decreases the immunogenicity of dendritic cells (16). Moreover, IDO expression leads to a rapid consumption of tryptophan in the tumor milieu (18). Below 0.5 μM tryptophan, T-cell proliferation is inhibited significantly (19). The reduction of tryptophan can also trigger stress-response pathways that respond to amino acid withdrawal, such as, general control non-derepressible 2 (GCN2) and mTOR. GCN2 responds to the presence of uncharged transfer RNA; amino acid insufficiency then activates GCN2, which leads to phosphorylation of its downstream molecule eukaryotic initiation factor 2α (eIF2α) (20). Phosphorylated eIF2α blocks the ribosomal translation of most mRNA species, and leads to cell-cycle arrest and functional anergy in CD8^+^ T cells (21). Activated GCN2 inhibits Th17 differentiation in CD4^+^ T cells but promotes immunosuppressive Treg differentiation and activity (22, 23). Thus, the depletion of tryptophan may impair anti-tumor response by inhibiting CD8^+^ cells function while promoting Treg activities. Targeting IDO-kynurenine-tryptophan axis could be an effective strategy to enhance the efficiency of immunotherapy. The IDO1-specific inhibitor 1-methyltryptophan has been shown to significantly inhibit IDO1 activity, and the effects of 1-methyltryptophan in enhancing T-cell responses against tumor antigens, allograft antigens, and autoantigens *in vivo* have been validated (24, 25). In addition, some orally available IDO1 inhibitors, epacadostat, and navoximod, have demonstrated safety and could reverse tryptophan depletion and kynurenine accumulation (26).

Similar to competition for tryptophan, competition for L-arginine also triggers GCN2 and mTOR signaling (27, 28). T cells with increased L-arginine levels display improved anti-tumor activity due to a combination of phenotypic changes, including improved survival and maintenance of a T central memory-like phenotype. However, some studies showed that arginine supports tumor cell growth and suppresses anti-tumor immunity (29). In tumor cells, L-arginine is a substrate of nitric oxide synthase and arginase (30). Additional studies indicated that nitric oxide activates cyclooxygenase-2 (31), which suppresses type I interferon–mediated tumor eradication in melanoma, and upregulates tumoral PD-L1 expression (32, 33). Thus, arginine metabolism in tumor cells promotes tumor progression and immune evasion. Depletion of L-arginine in leukemia by the addition of a PEGylated form of the catabolic enzyme arginase I (peg-Arg-I) has demonstrated anti-tumor activity (34, 35). However, L-arginine depletion also suppresses T-cell responses in tumors by inducing myeloid-derived suppressor cell (MDSC) infiltration (36). Therefore, therapeutic strategies that specifically deplete L-arginine metabolism in tumor cells are needed to eradicate tumor without dampening anti-tumor immunity.

Emerging evidence also shows that tumors may engage in high rates of fatty acid uptake. The survival and metastasis of tumor cells depend on fatty acid uptake and consumption, and subsequent catabolism through fatty acid β-oxidation pathway (37). Indeed, limiting low-density lipoprotein uptake reduces the oncogenic properties of pancreatic adenocarcinoma and renders cancer cells more sensitive to chemotherapy (38). Given that CD8^+^ effector T cells also take up fatty acids at high rates (39), fatty acid may be another environmental nutrient that the CD8^+^ effector T cells require to compete with tumor cells in the TME.

### Hypoxia

Hypoxia occurs when there is a shortage of dioxygen and tissues are inadequately oxygenated (40). In the TME, rapidly proliferation of tumor cells results in heterogeneously distributed zones of low oxygen concentration, which leads to hypoxic stress. Under such condition, hypoxia appears to be an important metabolic regulator that contributes to immunosuppression and tumor heterogeneity (41) (Figure 1).

Hypoxia can diminish anti-tumor immunity directly. One study showed that hypoxia abolishes the killing potential of natural killer (NK) cells by decreasing the surface expression of NK cell activating receptors NKG2D and CD16 (42). Hypoxia also induces T-cell apoptosis by inhibiting the expression of C-C motif chemokine receptor 7 (CCR7), which is essential for T-cell differentiation (43). Furthermore, while Treg infiltration in the tumor is associated with poor survival in patients with various cancers (17), hypoxia-activated hypoxia-inducible factor (HIF)-1 has been shown to promote Treg differentiation through upregulation of FoxP3 expression (44).

Hypoxia also suppresses anti-tumor immunity by upregulating immune checkpoint proteins. Tumor cells take advantage of this upregulation to suppress the anti-tumor function of immune cells through the interaction of the inhibitory costimulatory molecules with their ligands. Some studies showed that hypoxia-activated HIF-1α upregulates PD-L1 expression on tumor cells and immune cells by binding directly to a hypoxia response element in the proximal promoter of *CD274* (encoding PD-L1) (45–47). The upregulated PD-L1 limits cytotoxic T-cell activity, and thus increases the resistance of tumor cells to cytotoxic T cell-mediated lysis. As potential clinical applications, nitric oxide signaling could block hypoxia-activated HIF-1α function (48). Nitroglycerin (also called GTN), an activator of nitric oxide signaling, blocks PD-L1 expression in hypoxic tumor cells and suppresses hypoxia-driven cytotoxic T-cell apoptosis, thereby increasing the sensitivity of tumor cells to T cell-mediated cytotoxicity (45). As a mechanism to disrupt tumor hypoxia, the hypoxia-activated prodrug TH-302 also demonstrated pre-clinical benefits in improving immunotherapy efficiency by promoting CD8^+^ T-cell effector function and diminishing MDSCs (47). CD47, also known as integrin-associated protein, interacts with macrophages expressing signal-regulatory protein α (SIRPα) and delivers a “don't eat me” signal to avoid phagocytosis and innate immune surveillance (49). CD47 is overexpressed in various types of cancer, and its overexpression is correlated with poor prognosis in patients (50, 51). One study showed that HIF-1α-dependent expression of CD47 leads to decreased phagocytosis of tumor cells, which promotes cancer progression and immune evasion (52). MHC class I chain-related (MIC) molecules A and B both play important roles in tumor immunosurveillance and are expressed on NK cells, lymphokine-activated killer cells, and cytotoxic T cells (53). MIC molecules are expressed in various types of carcinomas (54), and their interaction with natural killer group 2D receptor on NK, LAK, and effector T cells leads to the activation of those cells and the subsequent lysis of tumor cells (55). One study revealed that hypoxia-activated HIF-1α decreases the surface expression of MIC molecules and leads to immune escape. The study also revealed that activation of nitric oxide signaling interferes with this immune escape mechanism (56).

In addition, hypoxia induces the secretion of immunosuppressive molecules in tumor cells. Through HIF-1α activation, hypoxia induces the expression of vascular endothelial growth factor (VEGF) (57), which promotes tumor angiogenesis. Also, tumor-secreted VEGF promotes the infiltration of MDSCs in the tumor (58), which inhibits the anti-tumor function of T cells and contributes to tumor progression (59). Secreted VEGF restricts the maturation of dendritic cells, blocks antigen presentation, promotes macrophage polarization from anti-tumor M1 to pro-tumor M2, and enables immune escape (58, 60). Thus, VEGF is a promising target for immune therapy. Other hypoxia-induced molecules, such as, CCL28, recruit CC chemokine receptor 10-positive Tregs to the tumor region and facilitate tumor immune evasion (61).

Collectively, hypoxia not only promotes an immunosuppressive TME but also induces the expression of immune checkpoint receptors and immunosuppressive molecules, leading to immune surveillance escape.

### Acidity

The TME of solid tumors is acidic because tumor cells favor aerobic glycolysis, which results in significant production of lactate and H^+^. With this metabolic preference, also known as the Warburg effect (62), the overabundance of lactate allows fast incorporation of carbon into biomass (e.g., nucleotides, amino acids, and lipids) and facilitates rapid cell proliferation (63). Although tumor cells maintain high production of H^+^, the major acidic metabolite is exported to the extracellular side. As a result, the TME becomes more acidic (64): the extracellular pH value inside patients' tumors is 6.9–7.0 (in contrast to 7.3–7.4 in normal tissues) (65), and in mouse tumor models, the tumor pH value is around 6.2–6.9 (66). Tumor acidity is important for tumor progression and metastasis (67) and is hypothesized to suppress the anti-tumor functions of immune cells (68) (Figure 1).

Acidic conditions have been found to inhibit the proliferation, differentiation, and cytokine production of cytotoxic T cells (69), and suppress their anti-tumor effects. However, the mechanisms underlying this process remain unclear. One possibility is that the pro-inflammatory cytokine IFN-γ is acid unstable and can be denatured in the acidic environment (70). IFN-γ is well-known to promote the recruitment of T cells through paracrine signaling. Notably, IFN-γ facilitates T cell-mediated killing by upregulating MHC class I expression on tumor cells and directly promotes tumor cell ferroptosis, a type of programmed cell death dependent on iron (70, 71). Furthermore, IFN-γ promotes the activation of M1 macrophages that are involved in anti-tumor immunity (72); assists in the differentiation of pro-inflammatory Th1; and inhibits pro-tumor Th2 differentiation (73). Thus, IFN-γ plays an anti-tumor role by maximizing the anti-tumor efficiency of M1 macrophages and CD8^+^ T cells. In turn, the denaturation of IFN-γ in the TME can suppress cytotoxic T cell-mediated tumor killing function and facilitate the polarization of the anti-tumor Th1 phenotype toward the pro-tumor Th2 phenotype, as well as prevent the activation of M1 macrophages (74). Therefore, in an acidic TME, tumor cells can easily evade immune surveillance and resist immunotherapy.

The negative impact of acidity on the anti-tumor activity of immune cells may manifest as clinical resistance. Indeed, studies showed that tumor acidity is correlated with poor prognosis in cancer patients who received ICI therapy (74, 75). Therefore, targeting acidity could be a promising strategy to improve immunotherapy efficiency. One option is to neutralize tumor pH by bicarbonate; after the pH value is neutralized in the TME, tumors showed improved response to ICIs as well as to adoptive T-cell therapy (76). Blocking the export of protons could also prevent tumor acidity. Tumor cells export intracellular protons by receptor enzymes, such as, V-ATPase. Esomeprazole, an inhibitor of V-ATPase, promotes the infiltration and effector function of IFN-γ^+^ cytotoxic T cells, suggesting that V-ATPase inhibition can increase the therapeutic potential of adoptive T cell immunotherapy (77). Collectively, the above findings indicate that tumor acidity induces immune evasion while inhibition of tumor acidity improves immunotherapy efficiency.

## Tumor Metabolites Promote Tumor Immune Evasion

### Adenosine

Adenosine is a product of the enzymatic breakdown of adenosine 5′-triphosphate (ATP), which can directly influence adenosine receptor-expressing cells and promote tumor growth, survival, and metastasis. Both adenosine and ATP are present at very low levels in extracellular fluids (78). However, ATP can be released into the extracellular milieu by cells under stress due to hypoxia, apoptosis, necrosis, etc. (79). ATP is gradually catalyzed and dephosphorylated by two ectonucleotidases, CD39 and CD73 (80), which generates adenosine (81). High expression of CD39 and CD73 is strongly correlated with poor clinical outcomes in patients with various cancer types (81–83). These ectonucleotidases can be induced by hypoxia and are highly expressed on cells in the TME, including tumor cells, MDSCs, Tregs, and tumor-associated macrophages (81, 84–86).

Extracellular adenosine activates signaling pathways through G protein-coupled adenosine receptors (AR), A1, A2a, A2b, and A3, all of which are widely expressed in both immune cells and tumor cells in the TME (87). Adenosine-AR signals enables tumors to escape immune surveillance by suppressing the activity of multiple anti-tumor immune cells, including CD8^+^ T cells, dendritic cells, natural killer cells, and M1 macrophages, while enhancing the activity of immunosuppressive cell types, including MDSCs and Tregs. The adenosine-AR signals in dendritic cells upregulate IL-10, IDO-1, TGFβ, and arginase-2, thus facilitating naïve T-cell differentiation toward Th2 lineages and promoting tumor growth (87). Adenosine also limits natural killer cell differentiation, proliferation, and production of the pro-inflammatory cytokines IFN-γ and TNFα (88, 89). In macrophages, adenosine induces pro-tumor M2 macrophage polarization by reducing the expression of IL-2, TNFα, and nitric oxide but upregulating arginase-1, IL-10, and VEGF (87, 90).

The adenosine-AR axis also promotes immunosuppressive cell functionality. One study showed that adenosine-AR promotes MDSC expansion and facilitates their immunosuppressive activity (91). A2BR activation on MDSCs and tumor cells promotes tumor progression by inducing VEGF secretion and angiogenesis (83, 92). Adenosine accumulation also boosts cancer cell survival and proliferation by activation of AKT, ERK1/2, JNK, and protein kinase C δc (93).

Unlike adenosine receptor A2AR, A2BR, and A3, one study showed adenosine receptor A1 (ADORA1) signaling axis suppresses tumor PD-L1-mediated immune evasion (94). Downregulation and inhibition of adenosine receptor A1 (ADORA1) significantly induces tumor PD-L1 expression by promoting ATF3 transcriptional activity. Although the role of adenosine signaling in tumor immunity is different from that in immune cells, the antagonist of the adenosine receptor 8-cyclopentyl-1,3-dipropylxanthine (DPCPX) has demonstrated synergistic effect with PD-1 antibody in melanoma and non-small cell lung cancer (NSCLC) (94).

**Figure 2:**
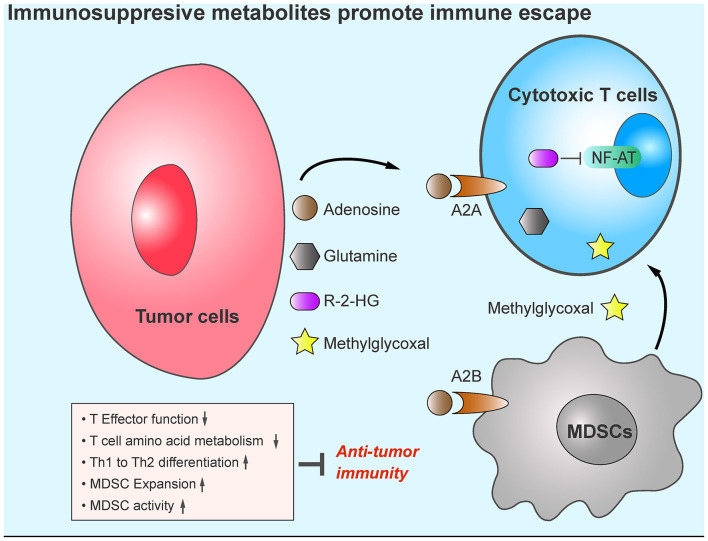
Tumor metabolites promote tumor immune evasion. Compared with other cell types, tumor cells display increased production of immunosuppressive metabolites, such as, adenosine, R-2-HG, glutamine, and methylglyoxal in the TME. These metabolites cause cytotoxic T cell anergy, increase the infiltration of MDSCs, and impair anti-tumor immunity.

Generally, adenosine exhibits a pro-tumor role in the TME (Figure 2). Therefore, targeting adenosine signaling pathways could be a promising anti-tumor strategy. CPI-444 is an orally available AR inhibitor; in a mouse model, it showed anti-tumor effect as a single agent and synergistic anti-tumor activity when combined with anti-PD-1/PD-L1 antibodies (95). Similarly, several AR antagonists in combination with anti-PD-1/PD-L1 are being investigated in the clinical trial stage (NCT02655822, NCT03207867, and NCT02740985). Depleting extracellular adenosine by blocking the activity of CD39 and CD73 could also restrict adenosine signaling pathways. Treatment with the CD39 inhibitor POM-1 or a blocking antibody demonstrated enhanced anti-tumor immunity by increasing cytotoxic T cell- and natural killer cell-mediated killing function in a mouse model (96). CD73 is in turn targeted by the monoclonal antibody MEDI9447, which inhibits CD73 ectonucleotidase activity. The combination of MEDI9447 and PD-1 antibodies in tumor models showed additive activity against adenosine-mediated immunosuppression, and a phase I study of MEDI9447 in cancer patients was initiated accordingly (NCT02503774) (97).

### R-2-hydroxyglutarate

Mutations in the *IDH1* gene (encoding isocitrate dehydrogenase 1) have been found in more than 70% of grade 2 and 3 astrocytomas and oligodendrogliomas, and in glioblastomas developed from these lower-grade brain tumors (98). The *IDH1* mutations occur arginine 132 in *IDH1* and arginine 172 in *IDH2* (99), and have been shown to induce the production of R-2-hydroxyglutarate (98). One study showed that tumor cells export R-2-hydroxyglutarate into the TME. The released R-2-hydroxyglutarate is taken up by activated T cells, where it significantly suppresses NFAT and NF-κB nuclear translocation and inhibits T-cell receptor signaling and polyamine biosynthesis, thus directly impairing T-cell activation (100) (Figure 2). Co-treatment with *IDH1*-mutant inhibitors (BAY-1436032 and AGI-5198) enhances the efficacy of immunotherapy against *IDH1*-mutant tumors *in vivo* (100–102).

### Glutamine

Glutamine is the most abundant free amino acid in the blood, and its circulating concentration is around 0.5 mmol/L (102). Glutamine plays an essential role in supplying both carbon and nitrogen sources for anabolic growth and proliferation (103). A recent study suggested that glutamine metabolic programing in tumor cells renders anti-tumor immune response less effective. Blocking glutamine metabolism by the glutaminase inhibitor JHU083 inhibits tumor growth by disabling “Warburg” physiology (104). JHU083 thus increases glutamine and glucose content in the TME. Although glutamine metabolism is also essential for CD8^+^ T cell activity (105) glutamine blockade shapes CD8^+^ T cells toward a highly proliferative, activated, and long-lived phenotype by upregulating glucose anaplerosis in CD8^+^ T cells (104). The role of glutamine metabolism in myeloid-derived suppressor cells (MDSCs) has also been reported; JHU083 reduces the recruitment of MDSCs to TME, induces MDSC apoptosis, promotes the conversion of MDSC to pro-inflammatory M1 macrophage, and renders ICI-resistant tumors sensitive to immunotherapy (106). Collectively, blockade of glutamine metabolism reshapes the TME and enhances anti-tumor immunity (Figure 2).

Emerging evidence indicates that extracellular glutamine promotes ferroptosis. One study demonstrated that glutaminolysis is essential for ferroptosis, and glutaminolysis inhibitor compound-968 prevents ferroptosis in mouse embryonic fibroblasts (107). Another study showed that miR-137 suppresses the expression of glutamine transporter SLC1A5 to decrease tumor cell glutamine uptake and inhibits ferroptosis, suggesting a critical role of glutaminolysis in promoting ferroptosis (108). However, ferroptosis is an important prerequisite of immune checkpoint therapy-mediated tumor eradiation (71). Thus, an inhibitor of glutaminolysis may suppress immune checkpoint therapy-induced ferroptosis. Detailed mechanisms of glutaminolysis and ferroptosis should be investigated to prevent this antagonism effect.

### Methylglyoxal

Methylglyoxal (MG), a by-product of glycolysis, is present ubiquitously in living cells (109–111). MG induces the production of advanced glycation end products and promotes tumor proliferation *in vivo* (112, 113). Because tumors exhibit high rates of aerobic glycolysis, enhanced aerobic glycolysis in tumors leads to the accumulation of MG in the TME, raising concerns about the role of MG in promoting immune evasion. One study indicated that MDSCs display high levels of MG production in the TME, which promotes the transfer of MG into cytotoxic T cells in a cell-cell contact-dependent manner and leads to the accumulation of MG in cytotoxic T cells. For the reason that MG can easily bind with L-arginine and L-glutamine, MG depletes these amino acids in those T cells and paralyzes anti-tumor immunity. N-N-dimethylbiguanide (DMBG), which neutralizes the glycation activity of MG, can reverse the suppressive effects of MDSCs on cytotoxic T cells and sensitize immunotherapy-resistant tumor to ICI treatment (114). Moreover, as an immunosuppressive metabolite, MG can be easily detected in peripheral blood. Therefore, it could serve as a biomarker of immunotherapy resistance (Figure 2). Although MG scavenger and ICIs have demonstrated encouraging synergistic therapeutic effects, the detailed mechanisms of how MG causes anti-tumor cell fatigue while facilitating the survival of pro-tumor cells need to be further investigated.

## Conclusion and Perspective

Although immune checkpoint blockade has demonstrated durable anti-tumor activity in a variety of cancers, the overall response rate is far from being satisfactory, necessitating a comprehensive understanding of the mechanisms of decreased anti-tumor immunity under a hostile TME and the identification of predictive biomarkers for patient selection. Tumor cells enhance nutrient uptake, deplete oxygen, increase acidity in the TME, and upregulate pro-tumor metabolite production to create an immunosuppressive TME, which promotes tumor progression and immune evasion. Therefore, targeting cancer cell metabolic pathways could restrict tumor growth and invasion as well as restore an anti-tumor TME. Also limiting to patient welfare has been the inadequacy of predictive biomarkers of response to immunotherapy. The metabolites secreted into the TME by cancer cells could be promising biomarkers owing to their accessible measurability. Thus, furthering our understanding of cancer metabolism will not only broaden the current knowledge of interplay in the tumor microenvironment but also overcome immunotherapy resistance by expanding therapeutic options so that more patients can benefit from immunotherapy.

## Author Contributions

ZJ collected the data and wrote the review. JH contributed to writing and discussion. YL and GH contributed to scientific discussion. M-CH edited this manuscript and supervised this work. All authors contributed to the article and approved the submitted version.

## Conflict of Interest

The authors declare that the research was conducted in the absence of any commercial or financial relationships that could be construed as a potential conflict of interest.
